# Extracellular Interactions of Alpha-Synuclein in Multiple System Atrophy

**DOI:** 10.3390/ijms19124129

**Published:** 2018-12-19

**Authors:** Dario Valdinocci, Rowan A. W. Radford, Michael Goulding, Junna Hayashi, Roger S. Chung, Dean L. Pountney

**Affiliations:** 1 School of Medical Science, Griffith University, Gold Coast 4222, Australia; dario.valdinocci@griffithuni.edu.au (D.V.); michael.goulding@griffithuni.edu.au (M.G.); 2 Centre for Motor Neuron Disease Research, Department of Biomedical Sciences, Faculty of Medical and Health Sciences, Macquarie University, Sydney 2109, Australia; rowan.radford@students.mq.edu.au (R.A.W.R.); roger.chung@mq.edu.au (R.S.C.); 3 Research School of Chemistry, Australian National University, Canberra 2601, Australia; junna.hayashi@anu.edu.au

**Keywords:** α-synuclein, multiple system atrophy, gliosis, glymphatic, neuroinflammation, chaperone

## Abstract

Multiple system atrophy, characterized by atypical Parkinsonism, results from central nervous system (CNS) cell loss and dysfunction linked to aggregates of the normally pre-synaptic α-synuclein protein. Mostly cytoplasmic pathological α-synuclein inclusion bodies occur predominantly in oligodendrocytes in affected brain regions and there is evidence that α-synuclein released by neurons is taken up preferentially by oligodendrocytes. However, extracellular α-synuclein has also been shown to interact with other neural cell types, including astrocytes and microglia, as well as extracellular factors, mediating neuroinflammation, cell-to-cell spread and other aspects of pathogenesis. Here, we review the current evidence for how α-synuclein present in the extracellular milieu may act at the cell surface to drive components of disease progression. A more detailed understanding of the important extracellular interactions of α-synuclein with neuronal and non-neuronal cell types both in the brain and periphery may provide new therapeutic targets to modulate the disease process.

## 1. Multiple System Atrophy and α-Synuclein

α-Synuclein (α-syn) is a small (14 KDa), acidic protein expressed in the brain, peripheral nervous system, platelets, megakaryocites, T cells, B cells, NK cells and circulating erythrocytes [1,2,3,4,5]. Its pre-synaptic localization and high abundance implicate an important role in synaptic transmission [6,7] with specific functions implicated in synaptic vesicle recycling and regulating soluble *N*-ethylmaleimide-sensitive factor attachment protein receptor (SNARE) interactions and dopamine biosynthesis [8,9,10,11,12]. α-Syn is also implicated in the control of dopamine release, re-uptake and pre-synaptic compartmentalization [13]. In vitro α-syn is a dynamically unfolded monomeric protein, although in vivo both monomeric and membrane-associated tetrameric, α-helical forms may be present [14,15,16,17,18]. Various factors, such as raised copper or calcium concentration, oxidative stress, and post-translational modifications, such as phosphorylation, can trigger intracellular α-syn aggregation [19,20,21,22,23].

**Figure 1:**
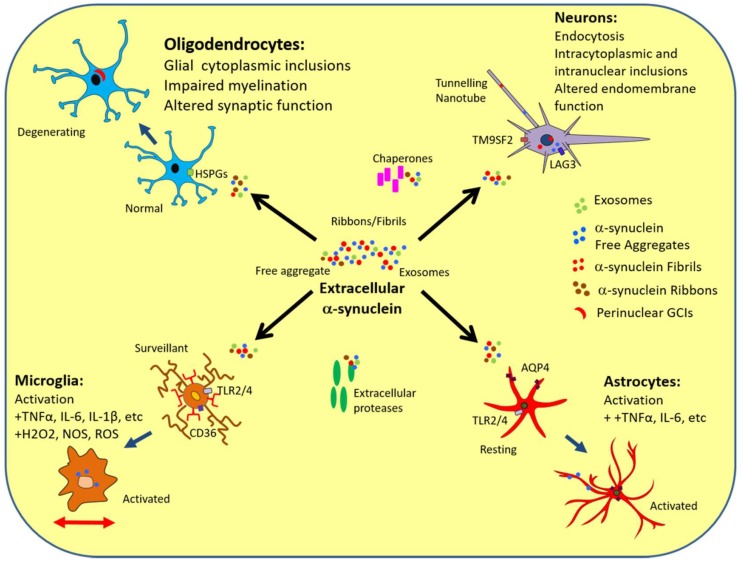
Interactions of α-synuclein with CNS cell types and the extracellular matrix in multiple system atrophy. α-Syn is released by neurons via either exocytosis or membrane leakage due to apoptosis, necrosis, or other damage. Neurons have LAG3 and TM9SF2 receptors on the surface which when bound by fibrillar α-syn, mediate clathrin-dependent endocytosis. Tunneling nanotubes can mediate α-syn transfer between various cell types. Released α-syn can interact with extracellular proteases and chaperones. Astrocytes detect α-syn and signal for microglial recruitment by inflammatory factors. This also has the effect of activating microglia from the surveillant state to the phagocytic phenotype. Activation is also caused when microglia detect α-syn either in exosomes or free in the extracellular matrix. Astrocytic activation could lead to loss of aquaporin-4 polarization to endfeet and dysregulation of glymphatic circulation. Oligodendrocytes can take up α-syn -containing exosomes from neurons via endocytosis and mediated by surface heparin sulfate proteoglycans (HSPGs). Microglia can engulf exosomes via macropinocytosis. Microglia perform phagocytosis on free and exosome-associated α-syn. Microglia undergo clathrin-mediated endocytosis as well as activation by CD36 scavenger receptor and toll-like receptors (TLRs) and can spread α-syn pathology by migrating away from the site of uptake.

Intracellular inclusion bodies composed largely of misfolded and/or aggregated α-syn are the defining neuropathological feature of several neurodegenerative diseases with complex Parkinsonian phenotypes, categorized as α-synucleinopathies, that include Parkinson’s disease (PD), multiple system atrophy (MSA) and dementia with Lewy bodies [24]. MSA is characterized clinically by autonomic dysfunction, including postural hypotension and urinary incontinence, and is distinct pathologically due to the presence of widely distributed α-synuclein-positive inclusions predominantly within oligodendrocyte cytoplasm (glial cytoplasmic inclusions; GCI) with occasional neuronal cytoplasmic and nuclear inclusions. α-Syn is primarily expressed pre-synaptically and oligodendrocytes and precursors express little α-syn mRNA either normally or in MSA, implicating uptake of extracellular α-syn secreted or released by neurons (Figure 1) [25,26]. Evidence exists of α-syn transfer from neurons to oligodendrocytes in vitro, further supporting that α-syn in GCI originates from neurons [27]. Multiple regions are characterized by neuronal loss and dysfunction, including putamen, substantia nigra pars compacta, pons, and cerebellum and show extensive reactive astrogliosis. MSA may initially be misdiagnosed as PD but has a shorter time-course of 6–9 years. Depending on clinical phenotype and α-syn distribution, MSA is classified either as MSA-P (Parkinsonism) or MSA-C (cerebellar ataxia) [28,29].

## 2. Secretion/Release of α-Synuclein

There is abundant data that α-syn is present in extracellular fluids. Raised concentration of oligomeric α-syn has been reported in blood plasma and cerebrospinal fluid (CSF) in α-synucleinopathy patients compared to healthy controls, indicating cellular secretion or release from dying cells [21], although total α-syn decreased in CSF (Table 1). The spread of α-syn pathology to grafted tissue was indicated to follow trans-synaptic release of oligomeric α-syn mediated by Hsp70 and DnaJ [30,31]. α-Syn oligomers can also be secreted from neurons via exosome vesicles that are in turn readily taken up via endocytosis [32]. Moreover, reduced autophagic degradation of oligomers and raised calcium both stimulated exosome-mediated release [33,34]. α-Syn oligomeric strains were found to spread more efficiently than fibrils and ribbons in vivo [35], with dopaminergic neurons found to perform trans-synaptic transport. Recently, microdialysis studies have shown that α-syn secretion is stimulated by neuronal activity and can be inhibited by blocking glutamate receptor activation, with α-syn release correlating with synaptic vesicle exocytosis [36]. Furthermore, glucocerebrosidase overexpression in vitro resulted in a significant decrease of exosome secretion of α-syn [37]. Indeed, the lipid peroxidation product 4-hydroxynonenal, a marker of oxidative stress in PD, could trigger the secretion of extracellular vesicles containing oligomeric α-syn species [38].

## 3. α-Synuclein Interactions with Neurons

The Braak staging of CNS spread of largely neuronal α-syn pathology in sporadic PD proposes migration from the olfactory bulb and medulla oblongata and subsequent spread to the substantia nigra, midbrain and basal ganglia [39]. MSA does not adhere to the Braak staging model, instead spreading via one of two pathways relating to the subtype present. In MSA-P, degenerative pathology occurs initially within the nigrostriatal region accompanied by α-syn deposits in the substantia nigra and putamen, later occurring in the frontal cortex [40]. MSA-C has been described as having a four-stage pathology, beginning in the cerebellar subcortical white matter and olivo-cerebellar fibers of the medulla and subsequently spreading to the basal ganglia, neocortex, amygdala, and hippocampus [41]. There is a regional correlation between GCIs and myelinated axons, with GCIs occurring predominantly in myelinating oligodendrocytes affecting the myelination of axons. In vivo cell-to-cell transmission showed various synthetic α-syn strains (oligomers, ribbons, and fibrils) have different pathogenicity profiles and uptake when administered locally or systemically [35]. Bolus CNS injection of synthetic α-syn fibrils into non-transgenic mouse caused movement dysfunction, neuronal aggregates, and neuronal loss [42,43], with migration of α-syn suggested to contribute to further inclusion body development [44,45,46].

Uptake of α-Syn by clathrin-mediated endocytosis has been demonstrated in vitro in neurons, oligodendrocytes, and microglia, although was not fully inhibited by blocking the pathway [27,47,48,49,50], suggesting the possibility of alternative routes of entry, such as caveolar endocytosis. Recently, neuronal lymphocyte activating gene-3 (LAG-3), was shown to facilitate specific α-syn endocytosis [51]. Furthermore, TM9SF2, a nonaspanin transmembrane protein associated with endosomes, has been linked to α-syn spread and cellular uptake [52,53]. Thus, α-syn endocytosis may in turn be associated with lysosomal dysfunction and may even provide a link to the various genetic loci linked to defective autophagy in α-synucleinopathy [54,55]. α-Syn selectively activates neuronal Cav2.2 channels and increases neurotransmitter release including dopamine [56].

α-Syn aggregates have been proposed to behave like prions, spreading from cell to cell by autocatalyzing the misfolding of endogenous α-syn [57,58,59]. Several studies have reported α-syn spread (and gliosis) in PD cases with fetal tissue grafts [60,61,62], indicating that pathological α-syn transfers from neurons to neighboring cells inducing α-syn aggregation in a process akin to prion diseases. This highlights the potential of extracellular α-syn to be taken up by CNS cells and spread neurodegeneration to anatomically adjacent brain regions. α-Syn fibrils are insoluble, and resist degradation in extracellular fluids, while the central hydrophobic region stabilizes the β-sheet conformation [25,63,64,65]. LB isolates induced neuronal α-syn pathology in macaque monkeys, with a later study demonstrating trans-neuronal transport of α-syn injected in mice [43,66]. Glial inclusion body formation was also reported in mice injected with dispersed GCI material from MSA patients [67]. Recently, it has been shown that SNCA knockout mice expressing human SNCA only in oligodendrocytes developed significant α-syn pathology in myelinating oligodendrocytes in fiber tracts after being inoculated with α-syn from synucleinopathies. Pathology developed the fastest following inoculations of MSA-derived α-syn; however, these mice failed to display any overt dysfunctional phenotype, and α-syn pathology subsided overtime [67]. Brain homogenates from MSA cases unlike homogenates from PD and DLB patients caused misfolding, inclusion formation and neurodegeneration in mice that express the human A53T familial PD α-syn mutant with α-syn aggregates observed primarily in neurons [59,68], indicating that α-syn in MSA is a different conformation to PD or DLB. It is interesting to speculate that pathogenic α-syn may be biochemically distinct in different α-synucleinopathies and may require additional factors, such as proteins, lipids, and nucleic acids, to exhibit prion-like properties. Indeed, recent studies by Woerman et al. (2018) have shown that MSA α-syn prion replication was selectively inhibited in cells expressing the E46K PD-linked α-syn mutation, but not other familial mutants [69]. This suggests that the glutamate to lysine substitution prevents the E46K mutant protein from being an appropriate template for prion replication, perhaps due to a functional change in α-syn caused by the replacement of a negatively charged amino acid to a positive charge.

## 4. α-Synuclein Interactions with Oligodendrocytes

Given that oligodendrocytes are not contiguous with neuronal synapses, GCIs in MSA are likely formed from α-syn absorbed from the interstitium. While MSA with long disease duration showed α-syn in astrocytic endfeet and Begmann glia, preferential uptake of α-syn by oligodendrocytes occurred in primary astrocyte/oligodendrocyte co-cultures [59,70,71,72,73]. While it is was observed that oligodendrial precursors express α-syn mRNA, the levels are not significantly elevated in oligodendrocytes from MSA patients compared to normal controls. This suggests that intrinsic production of endogenous α-syn by oligodendrocytes is not a significant factor in α-syn pathology [25,26]. Indeed, recent studies have shown that injection of MSA and PD-derived α-syn material from the CNS, but not peripheral α-syn pathological aggregates can induce disease pathology [73]. It has been demonstrated that cell surface heparan sulfate proteoglycans can mediate the entry of specific α-syn aggregate species [74,75]. Recent cell culture studies revealed insoluble fibrils bind and enter the neuronal and oligodendroglial cells via heparin sulfate proteoglycans; however, this was not the case in microglial or astroglial cells [75]. Soluble oligomers also used heparan sulfate proteoglycans; however, these were less efficient than the fibrils. Indeed, the current evidence indicates that a variety of mechanisms exist that mediate the entry of exogenous α-syn into cells. Further research is needed to evaluate if other potential endocytic sites or receptors exist that enable α-syn entry into oligodendrocyte cells or if therapeutic approaches can be developed to selectively inhibit uptake by unaffected cells. Furthermore, recent studies have implicated uptake and re-release of α-syn aggregates by oligodendrocytes in creating more pathogenic aggregate strains [67]. Whereas, accumulation of α-syn taken up by oligodendrocytes was shown not to be influenced by inhibiting macroautophagy [76]. Clearly uptake (and re-release) of α-syn by oligodendrocytes is of critical importance to the development and spread of pathology in MSA. A focus on understanding the mechanisms involved is crucial in the design of neuroprotective therapies. However, emphasis should also be given to understanding how α-syn acting at the cell surface influences intracellular signaling pathways and oligodendrocyte function.

## 5. α-Synuclein Interactions with Astrocytes

Astrocytes are involved in a wide array of functions including synaptic pruning, modulating neuroinflammation, and degradation of unwanted organelles [77,78]. Astrogliosis is characterized by cellular hypertrophy and progressive changes in gene expression, including upregulation of glial fibrillary acidic protein (GFAP), adhesion, antigen presenting, cytokine, growth factor, cytoskeleton, enzyme, trophic factors, and receptor proteins, such as peroxisome proliferator-activated receptors that are regulated by specific intra- and intercellular signaling [79]. There are two types of astrogliosis; anisomorphic reactive astrogliosis is irreversible, results in the formation of a glial scar and is associated with specific changes to cell growth, process morphology, proliferation, interdigitation, as well as increased GFAP, vimentin, and nestin. Isomorphic activation is transient and associated with stellate morphology, nuclear hypertrophy, and an increase in antioxidants and organelles, while growth factor secretion supports recovery [77]. Astrogliosis is a major component of MSA pathology and treating primary astrocytes with α-syn induces astrocyte activation by an ERK/MAPKK dependent mechanism [80,81]. Morphometric analysis of human cases and mouse models of MSA revealed that the degree of astrogliosis increased with proximity to GCIs [80,81], suggesting that extracellular α-syn may lead both to GCI formation via uptake and astrocyte activation by surface receptor interaction. Furthermore, oxidative stress treatment of transgenic mice overexpressing oligodendroglial α-syn led to astrogliosis associated with glial α-syn aggregates [82].

Because glial cells express little to no α-syn, glial uptake of α-syn or surface interactions may trigger the neuroinflammatory process, which may then operate in waves of incremental feed forward damage. Endocytosis was shown as a mechanism for direct uptake of α-syn by astrocytes from neuronal cell lines, which correlated with cytokine production (IL-1α, -1β, -6, -18), colony-stimulating factors and chemokines [83]. Astrocyte activation by α-syn was found to be mediated by the Toll-like receptor-4 (TLR-4) [84,85]. Indeed, α-syn-dependent activation of astrocytes resulting in upregulation of the secretory system may then combine with the inhibitory influence of endocytosed α-syn on endomembrane fusion leading to the accumulation of Munc18-positive vesicles observed in activated astrocytes in MSA [80]. Moreover, the temporal relationship between astro- and microgliosis in MSA is unclear as model studies do not reproduce the long duration of MSA pathogenesis. It is important to determine if astrocyte activation induced by extracellular α-syn then results in the recruitment of microglia due to the action of secreted factors. The prevalence of neuroinflammation, combined with the pro-inflammatory behavior of extracellular α-syn, implicates astrocytes in spreading MSA neuropathology as activated astrocytes may also secrete neurotoxic molecules.

CSF α-syn is decreased in MSA patient samples relative to controls ante-mortem despite an accumulation of pathological α-syn forms in CNS tissue and increased plasma levels [86]. Astrocytes play an essential role in the CSF circulation and removal of waste metabolites such as proteins by way of the relatively recently described glymphatic system [87]. Analogous to the lymphatic system, the glymphatic system functions to circulate nutrients and remove waste products through exchange of interstitial fluid with CSF. Some evidence suggests that the glymphatic system, in conjunction with astrocytes, may also be involved as a mechanism of α-syn dissemination in α-synucleinopathies. CSF passes into the perivascular space before exchanging with interstitial fluid in the brain parenchyma, a process that is driven by bulk flow because of differences in pressure and facilitated by aquaporin-4 transporters on the astrocytic endfeet that enclose the perivascular space [88]. Astrocyte activation has been shown to cause a perturbation of glymphatic function due to altered localization of the astrocytic aquaporin-4 transporter which is crucial in regulating glymphatic circulation, suggesting that astrocytic activation seen in MSA may lead to decreased clearance of α-syn via the CSF and contribute to pathology [89]. Although it has not yet been established that the glymphatic system can mediate α-syn clearance, other proteins, such as tau and β amyloid, that are prone to aggregation can be cleared by the glymphatic system in models of traumatic brain injury and Alzheimer’s disease [88,89]. Moreover, in a subset of MSA patients phosphorylated α-syn has been found in the endfeet of astrocytes at autopsy. While in a recent study a negative correlation was observed between astrocytic aquaporin-4 expression and α-syn deposition in the temporal neocortex of PD patients [90]. There may also be a correlation between the induction of reactive astrogliosis by extracellular α-syn that has been demonstrated in previous studies and abnormal aquaporin-4 distribution (Figure 1) [80]. Reduced levels of α-syn in the CSF of patients with α-synucleinopathies compared to neurologically normal and Alzheimer’s patients have been reported and this may in turn relate to impaired of α-syn clearance by the glymphatic system [91]. Novel functional glymphatic neuroimaging techniques may assist in evaluating the contribution of the glymphatic system in patients and in vivo models of α-synucleinopathies and contribute to our understanding of the role of glymphatic clearance dysfunction in the pathophysiology of α-synucleinopathies [92]. Further studies are needed to investigate if extracellular α-syn may directly affect aquaporin-4 expression or distribution in MSA and thereby modulate glymphatic clearance. Future experiments will need to focus on specific MSA animal and cell culture models as most experiments to date have used models of PD to address α-syn diseases, especially to elaborate the role of astrocytes in α-syn misfolding/aggregation and spreading.

## 6. α-Synuclein Interactions with Microglia

Representing about 10% of total brain cells, microglia, the principal immune-phagocytic CNS cell-type are derived from myeloid macrophages that migrate to cerebral regions during development, predominantly in the basal ganglia, hippocampus, and substantia nigra [93,94]. Resident microglia normally adopt a resting (surveillant) phenotype in non-overlapping domains of the brain, which is maintained by feedback of neuronal fractalkine, astrocytic glial derived neurotropic factor and other signaling molecules [95]. Disturbed homeostasis triggers microglial activation into M1 and M2 effector phenotypes, both of which are phagocytic, via interaction of stimulus with immune response receptors including complement factors, pattern recognition receptors and scavenger receptors [96,97]. M1 microglia secrete cytotoxic molecules and pro-inflammatory factors, such as TNF-α, IL-6, IL-1β, superoxide, NO, reactive oxygen species (ROS) and excitatory amino acids, and they can also mediate cellular damage and dysfunction [98]. Whereas, M2 microglia release anti-inflammatory IL-10 and transforming growth factor beta (TGF-β) and promote tissue repair via release of factors such as major histocompatibility complex 5, monocyte chemoattractant protein 1 and insulin-like growth factor 1 [99,100]. In primary mesencephalic neuron-glia culture systems, extracellular α-syn was shown to be phagocytosed directly by microglia resulting in microgliosis, upregulation of NADPH oxidase and secretion of ROS [101]. Elevated microglial activation in areas characterized by α-syn deposition and neurodegeneration was observed in patients with PD, DLB and MSA by positron emission tomography using a ligand that targets activated macrophages and has been confirmed by neuropathological studies of α-synucleinopathies [85]. Increased pro-inflammatory cytokines have also been observed in MSA cases [102,103,104]. Microgliosis can be identified around α-syn deposits years to decades after α-syn accumulation [85] or colocalizing with α-syn-rich neurons after direct stereotactic injection of α-syn ribbons or fibrils or dispersed GCIs [35,80]. Moreover, oligomeric α-syn induced a pro-inflammatory microglial phenotype by interaction with TLR1/2, leading to nuclear translocation of NF-κB and MyD88-dependent release of TNF-α and IL-1β [105]. However, the mechanism of α-syn induced activation of microglia remains unclear.

Due to their role in surveillance and in reaction to pathogens, microglia may exert an indirect effect on α-syn by secreting various toxic factors that disrupt the intracellular protein degradation machinery and thereby influence α-syn dynamics [106]. Moreover, neuron-glia cultures treated with lipopolysaccharide (LPS), a potent stimulator of microgliosis, showed increased H_2_O_2_-mediated chemoattraction towards α-syn [107], that increased by pre-injection of LPS in the rotenone mouse model [107]. Furthermore, localized microgliosis and astrogliosis was induced after 23 days by the injection of purified GCI material into the mouse medial forebrain bundle [80]. Although multiple factors contribute to the development of MSA the relative importance of which will likely vary from case to case, the overproduction of cytotoxic by-products by microgliosis may promote α-syn misfolding/aggregation.

Pathogen pattern recognition receptors in the microglial membrane permit identification of foreign structural motifs on pathogens but are also able to recognize changes in endogenous molecules, such as misfolded proteins, in neurodegenerative diseases [108]. In particular, toll-like receptors (TLRs) 2 and 4 are known to interact with α-syn. In a cellular model, purified microglial cultures from brains of wild-type (TLR4+/+) and deficient (TLR4−/−) postnatal mice were treated with wild-type and abnormal α-syn forms (fibrillary or truncated). This resulted in severe microgliosis in the TLR4+/+ groups, increased phagocytic activity, upregulation of nuclear factor kappa B (NF-κB), and increased production of CXCL1, IL-6, and TNF-α. Furthermore, TLR4-deficient microglia showed reduced production of ROS upon α-syn treatments. Moreover, both transgenic mouse models of MSA and human cases exhibit upregulation of TLRs [85,109,110]. This indicates that TLR receptors could be an important therapeutic target in MSA to ameliorate microglial activation by extracellular α-syn.

In vitro microglia can take up exosomes containing α-syn via macropinocytosis however are unable to effectively degrade fibrillar α-syn, yet vaccination of transgenic mice has been found to promote microglial clearance of α-syn [111,112]. Impairment of lysosome-mediated degradation of α-syn, the primary mechanism of degradation after phagocytosis, may give rise to the persistence of α-syn in microglia. α-Syn can also initiate receptor-mediated endocytosis in addition to macropinocytosis and clathrin/dynamin-mediated endocytosis to enter microglia facilitated by GM1-dependent lipid rafts [45,46,113], consistent with diverse α-syn uptake mechanisms. There is the potential for α-syn to attract and activate microglia that are then not capable of efficient clearance thereby leading to a prolonged pro-inflammatory reaction that could exacerbate neurodegeneration [96]. α-Syn can also recruit microglia to damaged cells by acting as a chemoattractant [114] and it has been shown to provoke both phagocytic and pro-inflammatory reactions in vitro via TLR-mediated pathways, such as TLR4, as well as via scavenger receptors, such as CD36 [80,85,96,114,115,116,117,118,119].

Microglia also display some characteristics that make them a potential vehicle for intercellular α-syn spread [99,120,121]. Microglial activation was observed to have migrated more rapidly than α-syn aggregates away from the site of injection after three weeks in mice injected with pathological α-syn aggregates [80]. Furthermore, the inability of microglia effectively to degrade pathological α-syn may predispose microglia to transport α-syn to adjacent brain regions as a component of disease progression. Recently, microglia were shown to take up α-syn aggregates and migrate away from the site of uptake without degrading the internalized protein, thereby mediating α-syn spread as an active transport vehicle. This potential mechanism of α-syn spread could be inhibited by the anti-cancer drug, epothilone D, that blocks tubulin depolymerization and microtubule dynamics [121]. Further animal model studies are required to determine if agents that block microglial uptake of α-syn or inhibit microglial migration could slow disease progression by reducing the microglial-mediated spread of α-syn pathology to anatomically connected brain regions.

## 7. Interactions outside the CNS

Many individuals affected by MSA have symptoms relating to a dysfunction of the autonomic nervous system (ANS). However, very little is known about the effects of α-syn pathology in this region. α-Syn has been reported throughout the ANS in PD, in particular the enteric nervous system (ENS), where it has been shown that α-syn is secreted and taken up by neurons as in the CNS [122,123,124,125]. Patients with PD often have non-motor symptoms preceding the onset of motor symptoms by over a decade, including olfactory disturbances [126], sleep disorders [127], autonomic dysfunction (especially constipation) [128,129], and depression [130]. Early symptoms combined with the presence of α-syn lesions in peripheral tissues such as the olfactory bulb, dorsal motor nucleus of the vagus (DMV) and the ENS [131] could have important pathogenic and clinical implications.

It has been hypothesized that α-synucleinopathies may originate in peripheral areas due to a neurotropic pathogen or other exterior insult and spread via cell-to-cell transmission of α-syn to the substantia nigra and other areas of the CNS [132]. This hypothesis started to gain traction when Braak and co-workers published a topographic distribution of LBs and Lewy neurites conducted on autopsy samples from patients with PD and incidental LB disease (ILBD). In all cases, the individuals were found to have α-syn -immunoreactive LBs and neurites in the DMV [39,133]. Using this information, the first iteration of the Braak staging system was devised whereby α-syn pathology manifests within the DMV before reaching the brain along the caudo-rostral axis [39]. Involvement of both the olfactory bulb and the DMV was explained by a proposed dual-hit mechanism involving both anterograde and retrograde progression leading to the brainstem [134].

α-Synucleinopathy originating outside the CNS has been the subject of several studies which have both supported and challenged the validity of the Braak staging system. Two such studies investigated the risk of PD in subjects who had undergone either a selective or full truncal vagotomy using data from Danish [135] and Swedish registries [136]. These studies found the risk for PD was significantly lower after a full truncal vagotomy compared to a matched general population cohort. However, this finding is contentious [137]. More recent retrospective autopsy studies have also shed doubt on α-synucleinopathies originating in the ENS, showing that many PD cases do not follow the predicted caudo-rostral distribution pattern within the brain, and often little to no DMV pathology is present. Therefore, the DMV is not an obligatory transfer or trigger site for α-syn pathology [138,139,140,141]. Furthermore, studies where α-syn aggregates isolated from affected peripheral neurons of PD patients were injected into rat brain have shown a lack of pathogenicity, suggesting that peripheral and CNS α-syn pathology may be unconnected co-phenomena [73].

Although there may not be a consistent staging system originating in the gut, there have been some connections discovered between the ENS and CNS sites of disease. Pathological α-syn injected into the stomach of rats is transported retrogradely from the gut to the brain via the vagal nerve [142] and overexpressed α-syn can also travel in the opposite direction [143]. Gut microbes from PD patients have been shown to exacerbate motor deficits in transgenic α-syn mice [144]. Gavage of mice with rotenone has even reproduced the progression of PD pathology in a gut to brain fashion [145], although this result has yet to be reproduced [146] and there are questions about the validity of the rotenone model [147]. More recently, removal of the vermiform appendix in early life was found to reduce the risk of PD in individuals living in rural areas, suggestive of a link to environmental toxin exposure transmitted via the gut. Indeed, α-syn was found to be expressed abundantly in healthy and PD appendix tissue [148]. MSA is characterized by a lower prevalence of gastrointestinal issues than PD, but there is an increased prevalence of genitourinary dysfunction. One study analyzed colonic biopsies from six MSA and 9 PD patients and aggregates of α-syn were detected in 5 out of the 9 PD patients but only in 1 out of the 6 MSA patients. Despite the small sample size and low frequency, this study did demonstrate that α-syn deposits can be observed in the ENS of those suffering from MSA [149]. Other areas of the ANS may be more prone to α-syn deposits, such as the pelvic nerve, which could help to explain the genitourinary symptoms experienced by many with MSA.

## 8. α-Synuclein and Extracellular Factors

Due to the differences in physical and chemical properties of the intra- and extracellular environment, (e.g., the extracellular environment is more oxidizing, exposed to ongoing sheer stress, lower levels of nucleotide phosphates and increased levels of calcium) the cell must possess different mechanisms to counteract toxic protein species, including α-syn, in the extracellular milieu [150].

Several proteases have been reported to degrade extracellular α-syn. The serine protease, neurosin, exists as an inactive, pre-pro-peptide intracellularly. Upon extracellular secretion, it converts into its active form upon the removal of the N-terminal pro-peptide [151]. GCIs in post-mortem tissue have been found to be immunopositive for neurosin [152], and neurosin can specifically cleave extracellular α-syn secreted from HEK293T cells. LC-MS/MS showed that neurosin mainly cleaves recombinant α-syn between Lysine 80 and Threonine 81 [153], located in the non-amyloid-β component region. Disintegration of this central amyloidogenic site of α-syn, may explain the neuroprotective effect of neurosin in MSA. This is further corroborated by neurosin-cleaved α-syn fragments inhibiting α-syn polymerization. Plasmin is a serine protease which solubilizes blood clots. In vitro, co-incubation of plasmin and recombinant α-syn resulted in cleavage mainly at theLys-Thr-Lys repeat region [154]. Using transmission electron microscopy and western blot analysis, Kim et al. reported that plasmin could also cleave oligomeric and fibrillar α-syn. Upon adding plasmin to a co-culture system between SH-SY5Y neuroblastoma cells overexpressing α-syn and a murine microglial cell line, BV-2, plasmin inhibited the neuron-to-microglia transfer of α-syn. Moreover, the addition of plasmin treated α-syn to astrocytes and microglia significantly decreased their activation, confirmed by a decrease in TNF-α and IL-1β expression [154]. Taken together, these findings emphasize the protective effect of plasmin on extracellular α-syn and progression of MSA pathogenesis.

Another class of proteases well-known to interact with proteins in the extracellular milieu are the calcium and zinc-dependent endopeptidases, matrix metalloproteinases (MMPs). MMPs regulate remodeling of the extracellular matrix by turning over proteins such as collagen and proteoglycans. During oxidative stress, a molecular hallmark of neurodegenerative diseases such as MSA, excessive upregulation of MMPs has been thought to promote further neuronal and glial damage [155]. Sung et al. treated dopaminergic SK-N-BE cells overexpressing α-syn with a nitric oxide generating compound, 3-morpholinosydnonimine. This increased the levels of α-syn secreted, and generated a partially digested, 6.5 kDa fragment of α-syn. When treated with an MMP inhibitor, no cleavage of extracellular α-syn was detected. An increase in MMP expression was concomitantly observed with an increase in both α-syn and nitric oxide levels. These fragments generated by MMP were significantly more cytotoxic, possessed a higher Thioflavin T fluorescence intensity, and were larger in size as confirmed by turbidity assays [156]. These results suggest that MMP may exacerbate the neurotoxic actions of α-syn in MSA.

It has been reported that protein chaperones released in the extracellular milieu can modulate the structure and toxic effects of extracellular α-syn. Hsp70, an intracellular molecular chaperone was found to be released into the extracellular environment upon heat shock treatment [157]. It is well established that Hsp70 can reduce α-syn toxicity and aggregation [158,159]. Danzer et al. found that when H4 glioma cells were co-transfected with α-syn and Hsp70, there was a 64% reduction in α-syn oligomers and Hsp70 in the extracellular media [30]. The same stress conditions were applied to Hsp70 transfected HepG2 cells, a hepatocellular carcinoma cell line. After culturing macrophages in the same media, these macrophages had a 7.5-fold higher TNF-α level compared to the non-Hsp70 exposed control [160]. This mechanism could exacerbate the activated, inflammatory state of microglia and astrocytes in MSA. Hsp90 has also been reported to interact with extracellular α-syn. Liu et al. stressed MES23.5 cells (a murine dopaminergic neuroblastoma fusion cell line) with MPTP and rotenone, followed by the addition of extracellular α-syn. A significant loss of neurites was observed when treated with both the stressor and extracellular α-syn. However, this was reversed upon treatment using geldanmycin, a Hsp90 inhibitor, suggesting a role of Hsp90 in modulating extracellular α-syn toxicity. Moreover, clusterin, an extracellular, ATP-independent molecular chaperone [161], has been shown to inhibit the fibril formation of α-syn at high levels in vitro [162]. In vivo, GCIs in oligodendrocytes have been found to be immunoreactive for clusterin [163]. Thus, chaperones may play a significant role in reducing extracellular α-syn mediated toxicity, and could in turn, slow the progression of MSA.

## 9. Conclusions—Implications of the Extracellular α-Synuclein Interactome

**Figure 2:**
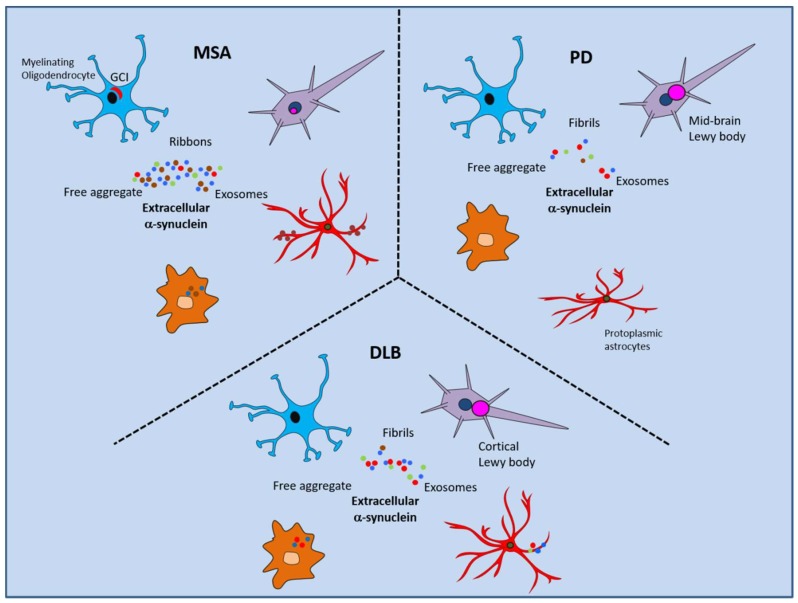
Differential interactions of extracellular α-syn in MSA, PD and DLB. Hypothetical scheme highlighting the differences in pathology that may arise due to variations in abundance, spread and strains of extracellular α-syn. All three diseases have reduced α-syn in CSF samples compared to controls and AD, indicating normal α-syn clearance is decreased or dysfunctional which might be due to altered glymphatic system and astrocytes. Microglial activation and clearance of α-syn by microglia is reduced in PD compared to MSA. Increased oligodendrocyte and astrocyte accumulation of α-syn by uptake in MSA suggests increased brain extracellular α-syn. Neurons show some α-syn pathology in MSA but this tends to be intranuclear and in areas related to neurodegeneration but also varies in cellular localization throughout the brain. Myelinating oligodendrocytes form GCIs whereas oligodendrocyte precursor cells do not. Whereas, non-myelinating oligodendrocytes develop α-syn pathology in PD but not until late stages. Oligodendrocytes may transmit α-syn between themselves along myelinating tracts. Protoplasmic astrocytes develop non-phosphorylated α-syn pathology in PD and are associated with a non-reactive, degenerative phenotype. Astrocytes are highly activated in MSA but α-syn accumulation is more correlated with age-related astrocyte gliopathy. α-Syn ribbons are taken up by oligodendrocytes and fibrils by neurons.

It is clear that a network of interaction sites exists between extracellular α-syn and each of the major CNS cell types and the extracellular matrix (summarized in Figure 1), with each interaction having the potential to contribute to secondary disease processes, such as neuroinflammation, synaptic dysfunction and cell-to-cell spread. Vehicles such as microglia and exosomes may mediate spread of α-syn pathology to distal brain regions. Each of the cellular interactions might be expected to occur in concert such that effective treatments will need to be multimodal. For example, gliosis involving increased activation of astrocytes and microglia could impede processes such as glymphatic clearance. Indeed, variations in α-syn strain and the differential spread and extracellular interactions may underlie the phenotypic differences in disease expression between α-synucleinopathies, such as PD, MSA and DLB (Figure 2). Recently, in α-syn inoculation models, Yun et al. (2018) used a GLP1R agonist to inhibit microglial activation and stop the conversion of astrocytes into a neurotoxic phenotype [164]. Thus, there are now three ways to inhibit neurotoxic astrocyte induction, by removing microglia via a CSF1R receptor agonist, by neutralizing the cytokines (TNF alpha, IL1b and C1q) that induce the neurotoxic astrocytes and by GLP1R agonist. Such approaches would have great potential in MSA as astrogliosis is more widespread than in PD. Overall, further studies will be needed to investigate novel approaches to ameliorating the various potentially important mechanisms of progression and toxicity of α-syn pathology and the various effects of the range of different cell types modulated functionally by interactions with extracellular α-syn.

## Figures and Tables

**Table 1:** Pathological distinctions between α-synucleinopathies. MSA may be distinct from PD and DLB due to the greater role of extracellular α-syn. There is increased sodium dodecyl sulfate soluble α-syn in MSA brain tissue [165], reduced retention of α-syn in neurons and uptake of α-syn by oligodendrocytes, astrocytes, and microglia.

	MSA	PD	DLB
**Biochemistry**			
*SDS soluble*	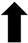	variable	n.d.
**CSF** **α** **-syn**			
*Total*	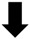	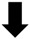	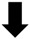
*Oligomeric*	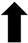	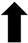	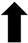
**Neurons**			
*Neuronal Loss*	+	+	+
*α-syn pathology*	±	+	+
**Astrocytes**			
*Gliosis*	+	+/− activation	Some activation
*α-syn pathology*	±	+	±
*Astrocyte subtype*	Subpial, ventricular and perivascular	Protoplasmic	Subpial, ventricular and perivascular
**Oligodendrocytes**			+/−
*α-syn pathology*	+	+/−	
*Oligodendrocyte subtype*	Myelinating	Non-myelinating	
*Pathological staging*	Primary event	Late Stage	
**Microglia**	Activation	Activation	Activation
**Prion Transmission**			
*Fetal grafts*	?	+	?
*Rodent models*	+	±	?
*Primate models*	?	±	?

## Abbreviations

α-syn	α-Synuclein
CNS	Central Nervous System
CSF	Cerebrospinal Fluid
DLB	Dementia with Lewy Bodies
GCI	Glial Cytoplasmic Inclusions
LB	Lewy Body
MSA	Multiple System Atrophy
MSA-C	Multiple System Atrophy-Cerebellar
MSA-P	Multiple System Atrophy-Parkinsonism
PD	Parkinson’s disease
SNARE	Soluble *N*-ethylmaleimide-sensitive factor attachment protein receptor
TLR	Toll-Like Receptor
LAG-3	Lymphocyte Activating Gene 3
